# Effects of adding early cooperation and a work-place dialogue meeting to primary care management for sick-listed patients with stress-related disorders: CO-WORK-CARE-Stress – a pragmatic cluster randomised controlled trial

**DOI:** 10.1080/02813432.2024.2329212

**Published:** 2024-03-31

**Authors:** C. Björkelund, E.-L. Petersson, I. Svenningsson, A. Saxvik, L. Wiegner, G. Hensing, I. H. Jonsdottir, M. Larsson, C. Wikberg, N. Ariai, S. Nejati, D. Hange

**Affiliations:** a Primary Health Care/Department of Public Health and Community Medicine, Institute of Medicine, Sahlgrenska Academy, University of Gothenburg, Gothenburg, Sweden; b Development & Innovation, Primary Health Care, Västra Götaland, Sweden; c Institute of Stress Medicine, Västra Götaland, Sweden; d Social Medicine/Department of Public Health and Community Medicine, Institute of Medicine, Sahlgrenska Academy, University of Gothenburg, Gothenburg, Sweden; e Institute of Medicine, Sahlgrenska Academy, University of Gothenburg, Gothenburg, Sweden; f Unit of Physiotherapy/Department of Health and Rehabilitation, Institute of Neuroscience and Physiology, Sahlgrenska Academy, University of Gothenburg, Gothenburg, Sweden

**Keywords:** Adjustment disorder/exhaustion disorder, stress-related mental disorder, randomised controlled trial, care manager, rehabilitation coordinator, primary care, days on sick-leave

## Abstract

**Objectives:**

To investigate whether intensified cooperation between general practitioner (GP), care manager and rehabilitation coordinator (RC) for patients sick-listed for stress-related mental disorder, combined with a person-centred dialogue meeting with employer, could reduce sick-leave days compared with usual care manager contact.

**Design:**

Pragmatic cluster-randomised controlled trial, randomisation at primary care centre (PCC) level.

**Setting:**

PCCs in Region Västra Götaland, Sweden, with care manager organisation.

**Participants:**

Of 30 invited PCCs, 28 (93%) accepted the invitation and recruited 258 patients newly sick-listed due to stress-related mental disorder (*n* = 142 intervention, *n* = 116 control PCCs).

**Intervention:**

Cooperation between GP, care manager and rehabilitation coordinator from start of illness notification plus a person-centred dialogue meeting between patient and employer within 3 months. Regular contact with care manager was continued at the control PCCs.

**Main outcome measures:**

12-months net and gross number of sick-leave days. Secondary outcomes: Symptoms of stress, depression, anxiety; work ability and health related quality of life (EQ-5D) over 12 months.

**Results:**

There were no significant differences between intervention and control groups after 12 months: days on sick-leave (12-months net sick-leave days, intervention, mean = 110.7 days (95% confidence interval (CI) 82.6 − 138.8); control, mean = 99.1 days (95% CI 73.9 − 124.3)), stress, depression, or anxiety symptoms, work ability or EQ-5D. There were no significant differences between intervention and control groups concerning proportion on sick-leave after 3, 6, 12 months. At 3 months 64.8% were on sick-leave in intervention group vs 54.3% in control group; 6 months 38% vs 32.8%, and12 months 16.9% vs 15.5%.

**Conclusion:**

Increased cooperation at the PCC between GP, care manager and RC for stress-related mental disorder coupled with an early workplace contact in the form of a person-centred dialogue meeting does not reduce days of sick-leave or speed up rehabilitation.

**Trial registration:** ClinicalTrials.gov Identifier: NCT03250026 https://clinicaltrials.gov/study/NCT03250026?tab=results#publications

CO-WORK-CARE

First Posted: August 15, 2017. Recruitment of PCCs: September 2017. Inclusion of patients from December 2017

## Background

Stress-related mental disorders are increasingly reported as reason for sick-leave [1]. In the Western world, total costs for work-related stress have been reported to amount to at least EU175 billion a year including sickness absence and productivity losses [2]. Detrimental work environment is a risk factor for sickness absence with these disorders [3,4]. A growing proportion of the diagnoses among common mental disorders (CMD) in primary care are stress-related disorders, i.e. adjustment disorder/exhaustion disorder (International Classification of Diagnoses (ICD) F43), today accounting for more cases of long-term sick-leave in Sweden than any other single diagnosis [1]. Stress-related mental disorders are a growing problem internationally, acknowledged in publications from the Netherlands, Germany, Switzerland, France, Sweden, United Kingdom, USA, Canada, and Australia [2,5]. In the Netherlands 47% of employers on sick leave now indicate stress as the main reason for absence [5]. According to a meta-analysis, there is evidence for associations between working conditions and near future burnout symptoms [6], as well as for increased risk of recurrent new episodes of sickness absence [6]. Work directed interventions in combination with clinical intervention have been reported to reduce time off work in a short perspective. However, in a literature review return to work (RTW) was the same both in the intervention and control groups with stress-related disorders at 12 months [7]. In a study of effects of a workplace dialogue, Karlsson et al. showed positive effects on long-term RTW for persons sick-listed because of severe stress, at least for younger intervention participants [8,9]. Positive effects of a Convergence Dialogue Meeting on work ability have been shown for patients with acute/subacute neck and back pain in primary care [10].

A knowledge gap still exists concerning vocational rehabilitation for people with stress-related mental disorders (adjustment and exhaustion disorder). This is especially relevant for primary care, where most sick-leave certifications with stress-related mental disorders are carried out. Care and rehabilitation of these patients represent a sigificant portion of the workload for primary care GPs, as well as for nurses, rehabilitation coordinators, psychologists and counsellors [11,12].

As in the entire CMD complex, stress-related mental problems affect working women to a greater extent than men [1]. The reason for this gender difference is not fully understood. A recent review of Nordic research showed that gender differences in sickness absence with psychiatric disorders mainly were explained by factors related to women and men working in different labour market sectors [3,6]. Female dominated sectors have more detrimental work environments in relation to CMD [13].

Primary care is the care level where the largest proportion of individuals with mental illness seek and also receive care [12,14]. Studies both from Sweden and from other countries in Europe and USA provide international evidence of the benefits of an organisational change to a collaborative care model called ‘care manager’ for the coordination of care for patients seeking care for mental illness [12,15]. Thus it has been shown that the care manager model positively affects the course of illness and the patient’s ability to return to work [15]. In a Swedish RCT, conducted in the primary care context, a significantly increased return to work (RTW) was shown for the patients who had contact with a care manager during a depressive episode compared to treatment as usual [15]. Patients stressed the structured continuous contact and support from the care manager as a ‘life line’ [16]. The care manager provides accessibility and continuity as well as psychopedagogic support to the patient and in a team-based care model facilitates access to a psychotherapist and other competencies at the PCC [15,17]. So far, studies have only been conducted including people with depression and anxiety syndromes [12].

Nieuwenhuijsen and coworkers concluded that a combination of a work‐directed intervention and a clinical intervention probably reduces the number of sickness absence days in patients with depression [18] but it is unclear if this is the case for patients with adjustment disorder [19] and no studies are avaible for patients with exhaustion disorder studying this combination.

The present study (CO-WORK-CARE-Stress, part of Co-Work-Care trial [20]) was designed to add intensified cooperation and a workplace dialogue meeting to the management of persons sick-listed with stress-related disorders (adjustment/exhaustion disorder ICD-10 F43) in primary health care. The addition is expected to enhance an established collaborative care manager organisation (CMO) at the PCC by promoting increased cooperation between a care manager, a rehabilitation coordinator responsible for sick-listed individuals’ return to work, and a general practitioner (GP) from the onset of illness notification [20]. The CMO and improved collaboration were coupled with an early person-centred dialogue meeting following the model presented by Karlsson et al. involving the patient and the employer, facilitated by the rehabilitation coordinator [21]. The intended mechanism to be tested through the addition of workplace dialogue was to provide more detailed information about factors at work that plausibly were reducing the patient’s work capacity. It also offered an opportunity for the sick-listed individual to describe and communicate in own words to the employer how the work situation contributed to experienced symptoms and reduced capacity.

## Aim

The aim was to investigate whether a structured intervention combining early cooperation between care manager, GP and rehabilitation coordinator combined with a person-centred workplace dialogue meeting led to earlier return to work and fewer net and gross sick-leave days over 12 months period in recently sick-listed patients with adjustment disorder/exhaustion disorder. The intervention was compared with the usual collaborative care manager organisation care [15].

## Methods

### Study setting and context

The CO-WORK-CARE-Stress trial was accomplished during 2017-2020 as a part of the CO-WORK-CARE trial [20]. All participants in the CO-WORK-CARE trial with diagnosis ICD F43 as reason for sick-leave are included in this sub-study CO-WORK-CARE-Stress trial.

In Region Västra Götaland an implementation of the care manager function at around 180 of the region’s 200 PCCs was accomplished in 2014-16. The implementation was a means for the region to increase the quality of care for patients with CMD at the primary care level. The care manager (usually a nurse) has an academic education (7.5 higher education credits) and as part of the regular duties works as a care manager (25-50%, around 10-20 h/week). The care manager’s primary responsibility is to establish a continuous contact with the patient and to provide psychopedagogic care and support the patient *via* regular telephone contacts during at least 3 months after the initial visit [12,15]. The care process is based on a person-centred 1-h initial meeting with the patient, focusing on addressing the patient’s needs, problems and requirements, plus the continuous contact which also entails regular self-assessments with validated self-assessment instruments. The care manager monitors the patient’s needs and facilitates contacts with a psychologist, physiotherapist, and/or counsellor when the patient’s condition permits or indicates need of additional treatment and care (psychologist consultation in around 40-50% of cases, physiotherapist 10%, counsellor 20-30% [15,22]). Contact with the GP is obviously more frequent depending on need of sick certification, ­medical examinations and initiation of rehabilitation ­process (see Table 1).

**Table 1.** Care process in the Co-work-care trial.

	GP	Care Manager	Psychologist, psychotherapist, counsellor, occupational therapist, physiotherapist	Rehabilitation coordinator	Dialogue meeting
Action	Diagnoses	Continuous contact from start of GP’s sick-leave certification	Contact in a stepped care procedure	Initiates return to work	Within 12 weeks
First contact 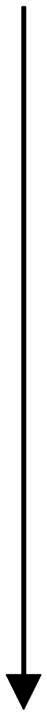	Sick-leave certification >14 days with adjustment/exhaustion syndrome diagnosis (F43). Somatic examination Information to care manager (and other staff when assessed needed)	Care plan together with patient based on interview and anamnesis. Assessment with KEDS MADRS-S GAD7 Psychopedagogic support	From first contact care plan/ team discussion: therapist contact based on patient’s diagnosis and needs	Interview with patient when care manager indicates patient ready to start rehabilitation process. Time for dialogue meeting agreed upon with patient. Dialogue meeting decided.	Person-centred dialogue meeting between patient and employer; Rehabilitation coordinator as the dialogue facilitator. Patient explains her/his view on return to work process
Follow-up	Evaluates need of further sick-leave in regular reappointments	Telephone follow-up week 1,2,4,8,12 When patient ready: work-related issues discussed	During illness course contact/ team discussion: therapist/other contact based on patient’s diagnosis and needs	Telephone contact with employer for interview about patient’s work situation and information about dialogue meeting scope	
Ongoing	*Structured cooperation between GP, care manager and rehabilitation coordinator*				

Grey parts common for control and intervention PCCs, green parts solely for intervention PCCs.

Since 2014 an implementation of assistant for coordinating the sick-leave and workplace rehabilitation process (usually named ‘rehabilitation coordinator’) was carried out at all PCCs in the region. A rehabilitation coordinator typically possesses knowledge in social insurance regulations and vocational rehabilitation and can serve as a regulation knowledge broker for the PCCs and a support for the patient’s RTW process and workplace cooperation. Unlike care managers, who are integrated into the regular personnel of the PCCs, the rehabilitation coordinator often serves multiple PCCs. The rehabilitation coordinator is usually engaged only for patients with long-term sick-leave duration (mostly >3 months). At some PCCs enrolled in this trial the care manager also served as rehabilitation coordinator (at 36% intervention PCCs, 42% control PCCs) [20].

### Trial design

The design was a pragmatic cluster-randomised controlled trial with complex intervention. The randomisation was performed at PCC level. The study adheres to Consort guidelines for pragmatic trials. The study was approved by the Swedish Ethical Review Authority.

### Participants

**Figure 1.**
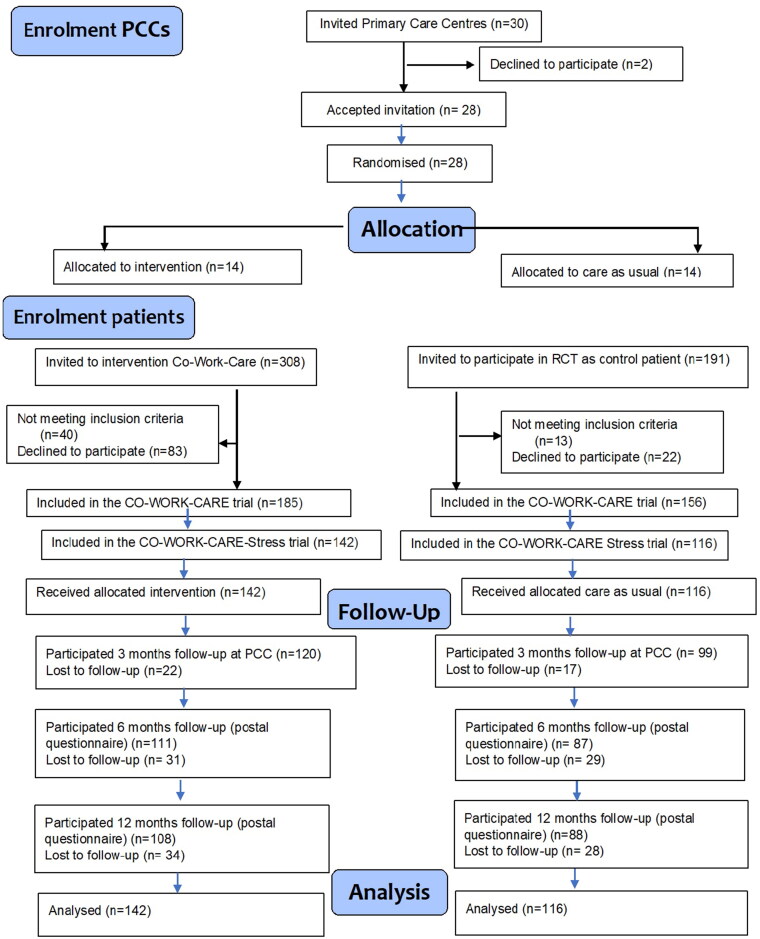
Flow chart CO-WORK-CARE-Stress trial as part of the CO-WORK-CARE trial.

Invitation to PCCs to participate in the trial started in September 2017. *via* support from the R&D Primary Care Region Västra Götaland, 30 PCCs in the region with an organisation consisting of a care manager and with affiliated rehabilitation coordinator were invited to take part in the CO-WORK-CARE RCT trial [20]. Many of the region’s PCCs that had a care manager organisation were engaged in other research projects, and the R&D organisation generally recommended that only one research project should engage the PCC at the same time. All PCCs, intervention as well as control, should also on their staff have not only a care manager (=part-time nurse) but also a psychologist/psychotherapist, GP, and nurse, as well as access to a rehabilitation coordinator. The PCCs should also be representative concerning public and private organisations, and from all parts of the region, as well as urban/rural localisation. Of the invited 30 PCCs, 28 (93%) accepted the invitation and participated in educational activities concerning the study protocol (flow chart Figure 1, upper part).

### Randomisation

Randomisation was conducted at PCC level. A total of 28 PCCs were included and divided into two strata, rural (*n* = 14) and urban (*n* = 14). The PCCs in each stratum were then divided into seven blocks of two PCCs each. One PCC in each block was randomised to the intervention group (*n* = 14) and one to the control group (*n* = 14) by the Primary Health Care R&D Region Västra Götaland, Sweden (flow chart, Figure 1). Cluster randomisation was chosen to avoid the treatment contamination between the intervention and control groups that would occur if patients at participating PCCs were individually randomised.

### Masking

No masking was possible, neither concerning personnel at PCCs nor research personnel.

### Patients

Patients attending the intervention and control PCCs, aged 18 and above, who were judged by a GP to need a completely new certification of illness due to incapacity to work related to the appearance of symptoms diagnosed as stress-related illness (adjustment disorder/exhaustion disorder ICD-10 F43; excluding post-traumatic stress disorder ICD F43.1) were invited by the GP or the care manager to participate in the trial. Diagnosis was established by the GP and recorded *via* the sickness certificate (main or second diagnosis). The diagnosis was confirmed by use of the PRIME-MD diagnostic instrument [23] and the Karolinska Exhaustion Disorder Scale (KEDS) [24].

### Exclusion criteria

Patients diagnosed with severe depression (Montgomery-Asberg Depression Rating Scale-Self (MADRS-S) ≥35) [25] or with medium to high risk of suicide (MADRS-S question 9 > 3), bipolar disorder, psychosis, addiction, pregnancy > 1^st^ trimester, cognitive decline, post-traumatic stress syndrome or not speaking/understanding Swedish were excluded.

### Intervention

The CO-WORK-CARE complex intervention consisted of: (i) structured cooperation between care manager, rehabilitation coordinator and GP from the initiation of sick certification and (ii) a person-centred dialogue meeting between patient and employer (immediate supervisor) with the rehabilitation coordinator serving as facilitator [20].

The patient’s care was based on the assessment and consultation by the GP concerning care and diagnosis of sick-leave, the individual care plan (which the care manager and the patient initially drew up together during the first 1 h face-to-face meeting), and course of symptoms, assessed by the patient, in the regular telephone contacts with the care manager. The individual care plan was drawn up between care manager and patient in both intervention and control arms [20]. However, at intervention PCCs the rehabilitation coordinator was included from the start, and the care manager, the rehabilitation coordinator and the GP maintained continuously communication about the course of the patient’s illness and progress, see Table 1. The intervention also consisted of a person-centred dialogue meeting between the employer and employee with the rehabilitation coordinator as the guide and facilitator, effectuated within 3 months from inclusion, as early as possible based on the patient’s opinion and the judgment of the GP and care manager. At both intervention and control PCCs the patient also had psychologist, counsellor, occupational therapist and/or physiotherapist contact in a stepped care model according to the patient’s illness course and needs in agreement with the ‘usual’ care manager function [15,17]. All the competencies at the PCC were thus potentially engaged in the patient’s care when deemed appropriate in agreement with the collaborative team care in the same way at all PCCs, both intervention and control PCCs.

The CO-WORK-CARE intervention with early structured cooperation between care manager and rehabilitation coordinator and GP, plus the person-centred dialogue meeting with the employer, was the added value in the intervention. The person-centred dialogue meeting was designed to make it possible for the patient to communicate to the employer her/his own view about possible factors that could facilitate RTW. The dialogue meeting was a simplified dialogue meeting, adapted to primary care from the more extensive protocol by Karlson et al. [21]. With the ambition to utilise as much as possible from the Convergence Dialogue Meeting principles it needed adaption to primary care clinical context with reduced resources for rehabilitation activities and with the overarching aim to focus on the needs of the patient in the RTW process.

### Preparations

#### Intervention PCCs

An 8 h session for the PCC’s personnel involved in care of patients with stress-related mental disorder (including the rehabilitation coordinator) to inform about the CO-WORK-CARE complex intervention was conducted for all intervention PCCs (more extensively earlier described [20]). The intervention PCCs were regularly supported by the research team to secure adherence to the complex intervention. The Co-Work-Care intervention was added at the intervention PCCs to the ‘care as usual’ care manager organisation at the control PCCs, see Table 1.

#### Control PCCs

Care as usual consisted of the care manager function at the PCC with a care manager contact with the patient for 3 months [15]. The care manager function included an initial meeting between patient and care manager, where a care plan was established. During the next 12 weeks regular telephone contacts mainly with the care manager guaranteed accessibility and continuity for the patient. This was provided in addition to other therapeutic and/or pharmacologic treatments according to the patient’s needs [15,16]. The care manager care process is further described in the PRIM-CARE trial [20]. Control PCCs were also supported concerning patient recruitment and follow-up. Contact with a rehabilitation coordinator was often only made in case of long sick-list duration (usually >2-3 months).

### Patient and public involvement

A person suffering from stress-related mental disorder was involved in the conducting, analysing and reporting of this research.

### Outcomes

Primary outcome: Number of sick-leave days (collected *via* the register-based database Micro-Data for Analysis of the Social Insurance System (MiDAS)) calculated from baseline to follow-up at 12 months at group level (intervention vs control patients). Number of sick-leave days was expressed as gross sick-leave days and as net sick leave days (part-time sick-leave combined to full days).

Secondary outcomes: Proportion of patients who returned to full time work, number and proportion of patients on sick-leave at 3, 6 and 12 months. Patient-reported outcome measures (PROMS): Stress-related symptoms: KEDS [24], depressive symptoms: MADRS-S [25], anxiety symptoms: General Anxiety Disorder Scale-7 (GAD-7) [26], Health related Quality of Life: EuroQoL-5D (EQ-5D) [27] and Work Ability Score (WAS) [28,29] changes. Frequency of antidepressant therapy, frequency of psychotherapeutic and other contacts.

#### Self-assessment instruments used for patient-reported outcome measures

Symptom progression was measured by the Karolinska Exhaustion Disorder Scale (KEDS) [24], a self-rating scale with nine items for assessment of symptoms of exhaustion disorder. Each item is rated from 0 to 6 points, scale range 0-54. A total point level >18 indicates risk for exhaustion disorder, points ≥ 30 indicates high level of exhaustion.

Depression level was measured with the Montgomery Asberg Depression Rating Scale-Self (MADRS-S) [25]. This is a widely used self-assessment scale for depression symptoms, especially designed for intra-individual measure of symptom level during the lapse of illness. The nine items are rated from 0 to 6 points with a total of 54 points. Points 0-12 are classified as no depression, 13-19 as mild depression, 20-34 as moderate depression and >34 as severe depression. In the CO-WORK-CARE trial patients with <35 points were included; ≥35 implied exclusion.

Anxiety was measured by the General Anxiety Disorder Scale-7 (GAD-7) [26]. The GAD-7 consists of 7 items, rated from 0-3 points with a total of 21 points. A total of >4 points is classified as mild GAD, >9 as moderate GAD and >14 as severe GAD [26].

The EuroQoL-5D (EQ-5D) five-dimensional questionnaire (British tariff) was used to measure health-related quality of life (HRQoL) [27]. The scale measures health status in the following five dimensions: mobility, self-care, usual activities, pain/discomfort, and anxiety/depression.

Work ability was measured by the Work Ability Score (WAS) VAS scale [28,29].

Patient data concerning work, family, education, socio-economy, lifestyle, and medication was collected *via* study questionnaire collected at the PCC at inclusion (baseline) and *via* postal mail at 3, 6, and 12 months.

### Statistics

Standard statistical methods were used for descriptive statistics. Continuous variables were analysed by independent sample t-test or Mann-Whitney U test and categorical variables and frequencies by Pearson chi-square test. Linear mixed model analysis with repeated measures was used to compare means of intra-individual change in depressive, anxiety and stress symptoms, WAS and HRQoL scores between the intervention and the control group. The analyses were adjusted for cluster randomisation of PCC, repeated measures for every person and pre-specified variables age, sex, education and antidepressants at inclusion. The model included a repeated effect for ‘time’ for each ‘person’ and ‘centre’ and a random intercept for each ‘centre’. These analyses were done to control for correlation in repeated measures for each ‘person’ and ‘centre’ and to adjust for cluster randomisation of PCCs. Linear mixed model analysis was performed for comparison of total number of 12 months sick-leave days between intervention and control group and were adjusted for cluster randomisation of PCC, age, sex, education and antidepressants at inclusion. A random intercept for each ‘centre’ was included in the model adjusting for cluster randomisation of PCC. EMMEANS subcommand from Mixed model in SPSS was used to compare estimated marginal means between the intervention group and the control group for every time in the study. The statistical analyses were made using statistical software SPSS, version 29 and SAS, version 9.4. Statistical significance was set at *p* < 0.05.

### Power calculation

The power calculation was based on achieving power for the primary outcome variable gross and net sick leave days and originally calculated for the Co-Work-Care trial [20]. To achieve a power of 80% to detect a difference of 3 units between the two groups at a significance level of 10% (two-sided), 150 patients were required in each group. The underlying assumption was a standard deviation of 10 units, a within-group correlation of 0.4 and a within-cluster correlation of 0.1, i.e. a design effect of 1.9 to correct for having a cluster analysis.

## Results

A total of 258 patients with diagnosis adjustment disorder/exhaustion disorder (ICD-10 F43) as main or secondary diagnosis were included, 221 women (86%) and 37 men (14%), see Flow Chart, Figure 1.

Table 2 shows demographic data at baseline together with baseline mean levels of stress symptoms, depression and anxiety symptoms and HRQoL and WAS levels for the intervention and control group.

**Table 2.** Demographic data for total patient group, sick-listed with stress-related mental disorder (F43) as first or second diagnosis (*n* = 258).

	Total patient group		Intervention		Control	
	n	%	n	%	n	%
Women	221	85.7	128	90.1	93	80.2
Men	37	14.3	14	9.9	23	19.8
Employment status						
Working	249	97.3	138	97.2	111	97.4
Studying	1	0.4	0	0	1	0.9
In search of work/other	6	2.3	4	2.8	2	1.8
Hours of work						
Full-time	226	87.9	125	88.0	101	87.8
Part-time (25-75 % of full-time)	31	12	17	12.0	14	12.2
Family situation						
Cohabiting	190	76.3	109	78.4	81	69.8
Single	65	25.2	30	21.6	35	30.2
Country of birth						
Nordic Country	233	90.3	131	92.3	102	87.9
Outside Nordic	25	9.7	11	7.7	14	12.1
Educational level						
12 years	157	60.9	94	66.2	63	54.3
> 12 years	101	39.1	48	33.8	53	45.7
Physical activity						
leisure time sedentary	34	13.3	13	9.3	21	18.3
Smoking						
Yes	55	21.3	25	17.6	30	25.9
Alcohol high						
(AUDIT > 8 p)	13	5.8	8	6.5	5	4.9
Lower socioeconomic index	135	57.4	81	60.9	54	52.9
Antidepressant medication yes	68	26.4	37	26.1	31	26.7
Main sick-leave diagnosis						
Depression (F32, F33)	23	8.9	16	11.3	7	6.0
Anxiety syndrome (F40, F41, F48)	15	5.8	6	4.2	9	7.8
Adjustment disorder/ Exhaustion disorder (F43)	200	77.5	111	78.2	89	76.7
	Mean	SD	Mean	SD	Mean	SD
Age	42.0	11.0	44.4	10.8	39.1	10.4
KEDS	28.7	8.7	28.3	8.5	29.3	9.1
MADRS-S	21.9	8.0	21.3	8.0	22.6	7.9
GAD-7	11.5	4.8	11.03	4.6	12.0	5.1
EQ5D-index	0.56	0.3	0.58	0.3	0.54	0.3
WAS	2.52	2.4	2.35	2.3	2.73	2.5

Lower socioeconomic index: worker, student, lower office clerk.

Lacking answer Employment status:2 persons, Hours of work: 1 person, Family situation: 3 persons.

Physical activity levels: non-active (sedentary), active (walking ≥4 h/week), regular training (several times/week).

Main sick-leave diagnosis was adjustment disorder/exhaustion disorder in 78% of patients and secondary diagnosis in 32%. More than 75% of the patients had elevated levels on all three assessment instruments KEDS, MADRS-S, GAD-7, in both intervention and control group.

### Primary outcome - Number of sick-leave days (net and gross)

Twelve months number of net and gross sick-leave days showed no significant differences between intervention and control group controlled for gender, education, antidepressive medication, age and cluster effect. Net mean of 12 months sick-leave days was 110.7 days (SE 13.9; 95% confidence interval (CI) 82.6-138.8) for intervention and 99.1 days (SE 12.6; 95% CI 73.9 − 124.3) for control patients (n.s.). Gross mean of sick-leave days was 160.3 (SE 17.6; 95% CI 124.8-195.9) for intervention and 132.5 (SE 15.8; 95% CI 100.9 − 164.1) for control patients (n.s.).

### Secondary outcomes

Proportion of patients who returned to full-time work and number and proportion of patients on sick-leave at 3, 6 and 12 months are shown in Table 3. The table also shows number of patients in intervention and control groups with antidepressant use and psychotherapeutic, counsellor, rehabilitation coordinator, physiotherapy and GP contacts at 3, 6, and 12 months. There was a significant difference concerning rehabilitation coordinator contact and counsellor and GP contact at 3 months, with higher proportion of contacts in the intervention group. However, there was no significant difference in the proportion on sick leave. Proportion of psychotherapy, antidepressant therapy, occupational and physiotherapist contacts did not differ between intervention and control groups (Table 3).

**Table 3.** Number and proportion of patients who returned to full-time work, number/proportion of patients on sick-leave at 3, 6 and 12 months, as well as number and frequency of antidepressant use, psychotherapeutic contacts, counsellor contacts, rehabilitation coordinator contacts, occupational therapy contacts, physiotherapy contacts and GP contacts at 3, 6 and 12 months for intervention and control group.

	Baseline		3 months		6 months		12 months	
	Inter-vention	Control	Inter- vention	Control	Inter- vention	Control	Inter- vention	Control
	n (%)	n (%)	n (%)	n (%)	n (%)	n (%)	n (%)	n (%)
Proportion full RTW	–	–	50 (35.2)	53 (45.7)	88 (62.0)	78 (67.2)	118(83.1)	98 (84.5)
Proportion on sick-leave	100%	100%	92 (64.8)	63 (54.3)	54 (38.0)	38 (32.8)	24 (16.9)	18 (15.5)
Antidepressant use	37 (26.1)	31 (26.7)	40 (33.3)	37 (37.4)	35 (31.5)	31 (35.6)	32 (29.6)	29 (33.0)
Psychotherapy/ Therapist contact	–	–	62 (48.8)	62 (59.0)	44 (39.6)	31 (35.6)	19 (17.6)	12 (13.6)
Counsellor contact	–	–	**30 (25.0)**	**10 (10.1)**	16 (14.4)	6 (6.9)	6.0(5.6)	4.0 (4.5)
Rehabilitation coordinator contact	100%	100%	**131 (92.3)**	**66 (56.9)**	**15 (3.5)**	**5 (5.7)**	5 (4.6)	2 (2.3)
Occupational therapist	–	–	21 (17.5)	20 (20.2)	14 (12.6)	15 (17.2)	6 (5.6)	6 (6.8)
Physiotherapy contact	–	–	31 (25.8)	27 (27.3)	16 (14.4)	20 (23.0)	12 (11.1)	9 (10.2)
GP contact	100%	100%	**90.6**	**80.9**	74 (66.7)	48 (55.2)	39 (36.1)	29 (33.0)

Bold figures, statistically significant difference.

Comparison between the group of patients who had access to care manager and rehabilitation coordinator as different persons (17/28 = 61%; 9/14 intervention PCCs, 8/14 control PCCs) as opposed to the group of patients who had access to care manager and rehabilitation coordinator as the same person revealed no significant differences in the number of net sick-leave days at 12 months between intervention and control group (intervention 90.5 days vs control 98.9 days, *p* = 0.68). When the same person acted as care manager and rehabilitation coordinator, the net number of sick-leave days was higher in the intervention arm than when these roles were filled by different persons, with a larger difference in sick-leave days between intervention and control group: intervention 128.3 vs control 99.8 days. However, this difference was not significant when controlling for cluster randomisation, *p* = 0.37.

Not all patients in the intervention group had a person-centred dialogue meeting with the employer: 55% (*n* = 78) had a dialogue meeting within 3 months, but 45% (*n* = 64) did not have a dialogue meeting. One major reason was that the patient returned to work within before the meeting was arranged.

### Patient-related outcome measures

The course of mental health symptoms according to KEDS, MADRS-S and GAD-7, as well as health related quality-of-life (HRQoL) (EQ-5D) and work ability score (WAS), is shown in Figure 2. There were no significant differences between the intervention and control group concerning any of these measures. The groups of patients with high KEDS measure at baseline (>29) in intervention and control group did not show any significant differences (data not shown).

Figure 2.Course of stress (a), depressive (b), and anxiety (c) symptoms, as well as work ability (work ability scale/WAI) (d) and EQ-5D (e), for intervention and control group patients during 12 months from baseline (3, 6, and 12 months) in the CO-WORK-CARE-Stress trial. Mixed model analysis with means adjusted for clustering, age, sex, education, and antidepressants at inclusion.

**Figure:**
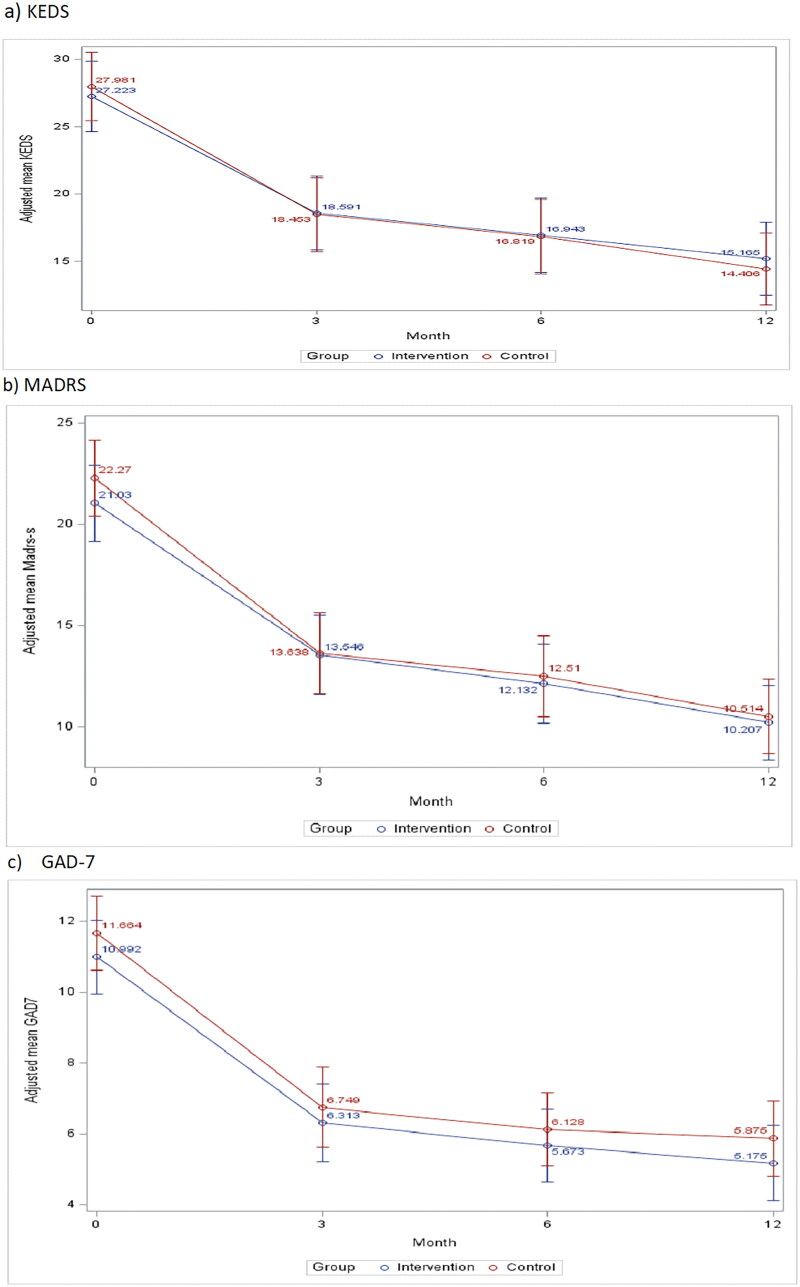

**Figure:**
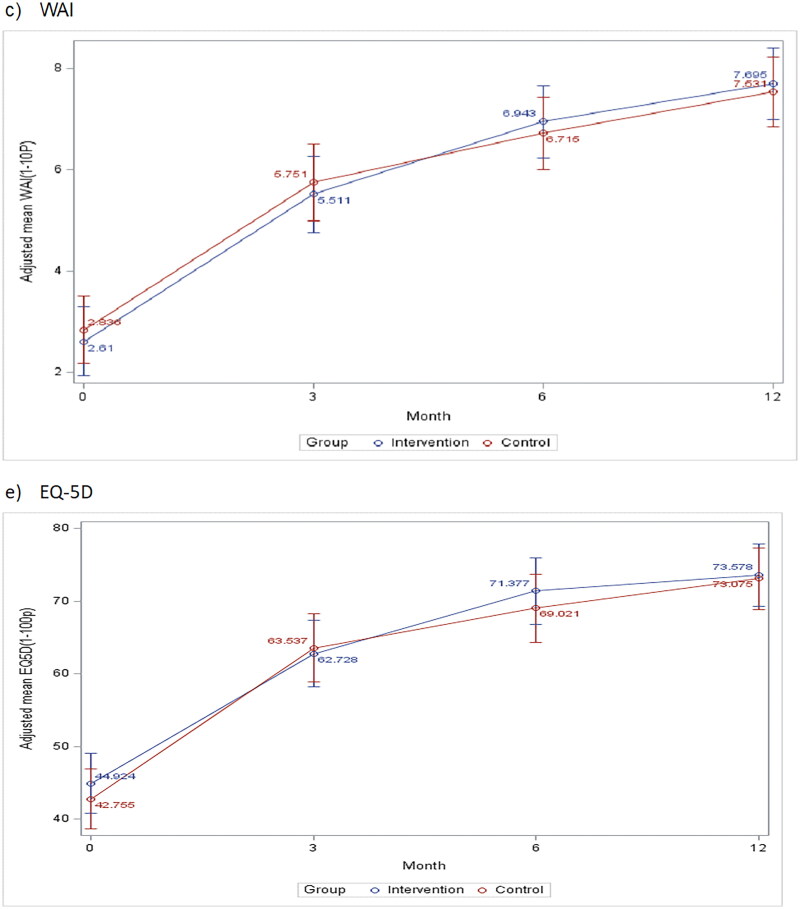

A high percentage of the 258 patients, 37.4%, reported high symptom levels on all three self-assessment instruments, identified as KEDS >29, MADRS-*S* > 20, and GAD > 9 at baseline (38% of the patients in the intervention group and 36% among those in the control group). Of the 196 patients who attended 12 months follow-up, only 0.5% in intervention and 2% in control group still reported high symptom levels on all assessment instruments at 12 months. Only 1 patient (belonging to the control group) deteriorated to high symptom levels on all three instruments.

#### Non-participant analysis

Baseline values for non-participants in the 12 month follow-up (*n* = 62) showed higher proportion of men in the non-participation group compared to 12 month participants (*n* = 196) (29.0% vs 9.7%, *p* < 0.001), lower educational level (university education 19.4% vs 45.4%, *p* < 0.001), higher proportion of lower socioeconomic index (71.9% vs 52.8%, *p* = 0.011), younger mean age (38.2 vs 43.2, *p* = 0.001) and lower mean KEDS level (25.8 vs 29.6, *p* = 0.003).

## Discussion

### Main findings

Structured early cooperation between care manager, GP and rehabilitation coordinator at the PCC combined with an early person-centred dialogue meeting between the sick-listed person and employer facilitated by the rehabilitation coordinator did not result in a reduction of sick-leave time compared with usual care which included a collaborative care manager organisation. Sick-leave time was consistently longer in the intervention group but controlling for cluster randomisation, which must be carried out in a cluster randomised trial, this difference in favour of the control group disappeared. Thus, adding additional early care contact plus structured person-centred contact with the workplace does not seem to influence either the course of sick-leave or symptoms.

Mean gross sick-leave duration was around 5 months for the intervention group and 4.4 months for the control group. Although not a statistically significant difference the results indicate that this group of patients with high stress levels and reduced work ability and in need of absence from work did not benefit from an early dialogue meeting with the workplace, even when conducted in a person-centred form. Thus, increased cooperation, care contacts and early contact with workplace does not seem to add to offering collaborative care with a care manager as the guarantor of good accessibility and continuity for the patent along with a stepped care adapted for the patient’s needs and desires.

### Other studies

The result is in line with results from other intervention studies [7,18]. The latest RCT studying integration of vocational rehabilitation and mental healthcare for patients with stress-related disorders showed even a negative impact on vocational outcomes compared to usual care [30]. In the trial, 71% had adjustment/exhaustion disorder and 29% ‘stress’. The ‘service as usual’ arm (a non-investigator-directed control group of standard mental healthcare and standard vocational rehabilitation) showed considerably faster RTW compared to vocational rehabilitation intervention. Long-term follow-up showed the same results [7]. In our study, around 85% of the patients had fully returned to work after 12 months, irrespective of belonging to intervention or control group.

A study of patients with stress-related exhaustion showed similar results as our study, that adding more extensive intervention to a standard treatment does not reduce sick-leave days or burnout symptoms [31]. The intervention consisted of individually tailored interventions; physical activity, CBT for insomnia/self-esteem, and/or cognitive training [31]. Other interventions comparing workplace dialogue and Acceptance and Commitment Therapy [32] could not show differences in sick-leave rates, and in a study of patients with adjustment disorder/exhaustion disorder no differences in sick-leave days could be shown when comparing work-focused CBT and CBT [33].

### Interpretation of findings

The development of today’s labour market with sectors of more detrimental work environment contributing to the development of stress-related illness has brought about a knowledge gap concerning prevention, best care and rehabilitation, both concerning work environment and the vocational and care sectors. Perhaps a more extensive occupational health services in cooperation with companies and the responsible workplace management could intensify both research and development of more health promoting workplaces and leadership [31]. In spite of increased efforts from primary care (and in some cases occupational health services where these exist) as in CO-WORK-CARE-Stress, increased cooperation and early contact with the workplace do not seem to reduce sick-leave duration or increase RTW.

One observation regarding the finding of this study is that many of the patients returned to work already after 3-6 months and as many as 70% were not on sick-leave at 6 months follow-up. Thus, adding more intensive support to the process does not seem to further enhance this RTW rate and the support already offered to the patients seems to be sufficient. Maybe the early introduction of contact with the workplace for the stressed patient might increase an already high stress level. This could be a possible explanation of why the number of sick leave days might be somewhat higher with the intervention, albeit not to the degree of reaching significance.

In usual care, the rehabilitation coordinator contact is often initiated first around after 2-3 months, either because the patient’s RTW is approaching or the RTW process is complicated. However, interviews with GPs and care managers and rehabilitation coordinators engaged in the CO-WORK-CARE intervention showed that the early cooperation around patients on sick-leave in addition to ‘usual care’ (care manager support including psychotherapeutic and other competence contact in a majority of cases in a team-based stepped care model) improved the often complex and frustrating work situation for the GP medically responsible for the care of the patient and the sickness certification process [34]. Interviewed GPs in the CO-WORK-CARE trial perceived that they gained better control and support for the care of the patient through the close collaboration around the patient [34]. This finding, together with the finding that having the same person serve as care manager and rehabilitation coordinator tended to prolong mean sick-leave time, implies that access to several and different competencies during the different stages of illness and recovery is important. The care managers and rehabilitation coordinators also appreciated the cooperation [35].

### Harms

There were no reports of harmful events, suicide attempts or psychiatric hospital care for the participants in the CO-WORK-CARE-Stress trial during the 12 months observation time.

### Strengths and limitations

The strength of this study is the RCT design involving several PCCs and different professionals. Inclusion of all patients seeking primary care for symptoms of stress coupled with reduced function and need of sick certification means that both medium and serious levels of stress-related adjustment/exhaustion disorder were included in the trial. Even if the study is very relevant for the primary care context, this could be a limitation if care and treatment of well-defined exhaustion disorder differs from ‘milder’ adjustment disorder. The group of patients with the most serious stress symptoms might have had need of a more intense dialogue intervention with several meetings.

More than half of the patients in the control group did report contact with a rehabilitation coordinator at 3 months, and even if this contact was not initiated from the start, this might have affected the outcome of the study as this part of the intervention does not entirely separate the two groups.

Further limitations might be that the totally 30 PCCs in the region recommended by the R&D organisation suitable for the Co-Work-Care intervention did not give full conditions for extensive randomisation regarding different aspects of the included PCCs. No follow up concerning workplace modifications was included in the study.

Another limitation was that almost 45% in the intervention group did not have a dialogue meeting, an important part of the intervention. However, 35% of the intervention group patients were back to work at 3 months, most of which were in the group that did not have a dialogue meeting. This means that the majority of patients with longer duration of sick leave did have a dialogue meeting.

A limitation was also that the number of included patients with stress-related mental disorder did not reach full power to compensate for cluster randomisation.

### Implications

The most important implication is that most patients with stress-related mental problems improve with time both regarding sick-leave and symptoms. This can be accomplished with relatively small resources, i.e. a collaborative care manager organisation and access to a rehabilitation coordinator when needed, securing continued support to the patient. Patients with adjustment disorder/exhaustion disorder do not seem to benefit from adding more intensified care. One aspect regarding the contact with the workplace could be that the timing of this needs to be considered. It’s important to emphasise that the study results concerns patients in primary care with a great span of different levels of stress-related disorder and with a primary care complex intervention during the earliest 3 months of an illness with sometimes much longer duration. Even if early and intensified cooperation between care manager, rehabilitation coordinator and GP plus an early workplace contact does not have more beneficial effects on the patients’ return to work, the cooperation brings support and aid for the personnel in the care of the patient.

### Concluding remarks

Our study of an intervention to decrease total time on sick-leave by initiating an early cooperation within the PCC as soon as the patient with stress-related mental disorder goes on sick-leave and including an early person-centred dialogue employer meeting did not demonstrate increased RTW or reduction in 12-month total sick-leave time compared to usual PCC care with ‘usual’ collaborative care manager organisation. Stress levels were almost identical at 12 months in both intervention and control groups, which highlights even more that patients with high stress-related symptom levels are not benefited by an early intensified RTW process.

It seems that offering continuous contact and accessibility to different therapeutic competencies combined with a team-based support system that consistently addresses the patient’s needs and initiates the RTW process when the patient is prepared for it may be the most effective and the best way of care.

## Data Availability

Data are not publicly available due to Swedish law but are available from the authors on reasonable request. The data are stored at the Gothenburg University/Allmänmedicinskt Centrum, Arvid Wallgrens backe 7, 40530 Göteborg, Sweden. Contact details: Cecilia Björkelund Orcid iD: 0000- 0003- 4083- 7342.
